# Relationship between high-density lipoprotein cholesterol levels and nutritional risk screening-assessment-intervention: a multicenter cross-sectional study

**DOI:** 10.3389/fnut.2025.1528068

**Published:** 2025-06-18

**Authors:** Qian Li, Hong Zhu, Xianghua Ma, Yan Zhao

**Affiliations:** ^1^Department of Clinical Nutrition, Suqian Hospital Affiliated to Xuzhou Medical University, Nanjing Drum Tower Hospital Group Suqian Hospital, Suqian, China; ^2^Department of Clinical Nutrition, Jiangsu Province Hospital, The First Affiliated Hospital of Nanjing Medical University, Nanjing, China

**Keywords:** high-density lipoprotein cholesterol, multicenter study, nutritional risk screening (NRS2002), nutritional assessment, nutritional intervention

## Abstract

**Background:**

Globally, there is limited literature exploring the relationship between nutritional risk screening, nutritional assessment, nutritional intervention, and HDL-C levels. This study analyzes the relationship between HDL-C levels, nutritional risk screening, assessment, and intervention among newly admitted patients in Jiangsu Province.

**Methods:**

Between October 2020 and June 2021, this study randomly selected 23 hospitals from 12 cities in Jiangsu Province using a stratified cluster sampling method. For nutritional assessment, the study used NRS2002 for risk screening.

**Results:**

4,190 patients were assessed, revealing a low HDL-C prevalence rate of 30.7%. The prevalence exhibited an “N” shaped distribution with age. The prevalence of low HDL-C among patients assessed at nutritional risk was 34.6%, 1.228 times higher than that of patients without nutritional risk. In terms of nutritional assessment, patients with constipation, severe infection, chronic kidney disease, fever, high CRP, and hypoalbuminemia significantly increased risks of low HDL-C by 1.432, 2.496, 1.543, 3.056, 1.794, and 2.703 times, respectively. Patients with a history of esophageal stricture, malignant tumors, and closed head injuries reduced the risks of low HDL-C by 60.9, 23.3, and 78.8%, respectively. Additionally, patients with nausea and vomiting, pancreatic insufficiency, severe infection, fever, and hypoalbuminemia decreased HDL-C levels by 0.156 mmol/L, 1.465 mmol/L, 0.403 mmol/L, 0.301 mmol/L, and 0.250 mmol/L, respectively. Regarding nutritional intervention, compared to patients who did not receive intervention, those receiving parenteral nutrition significantly lowered HDL-C levels at 1.014 mmol/L, with an increased risk of low HDL-C by 2.048 times. All *P*s <0.05.

**Conclusion:**

Nutritional risk, nausea and vomiting, constipation, pancreatic insufficiency, severe infection, chronic kidney disease, fever, high CRP, hypoalbuminemia, and receiving parenteral nutrition are associated with lower HDL-C levels in patients. A history of esophageal stricture, malignant tumors, and closed head injury is associated with higher HDL-C levels in patients.

## 1 Introduction

In recent years, the significant rise in the incidence of chronic diseases, driven by social and economic development and lifestyle changes, has posed a severe challenge to the public health system. Globally, chronic illnesses like cancer, diabetes, and cardiovascular disease are now among the main causes of morbidity and death. These illnesses are a major focus of international public health studies and interventions because they not only have a significant negative influence on an individual’s health but also use a significant amount of medical resources.

Nutritional status has received significant attention as a crucial factor affecting chronic diseases’ start, course, and prognosis. Nutrition is essential in the prevention and management of chronic diseases. Malnutrition, whether undernutrition or overnutrition, can exacerbate disease progression and worsen patient outcomes. Nutritional risk screening and evaluation accurately identify patients with malnutrition or those at risk of nutritional deficiencies. They greatly aid in the prompt detection and treatment of nutritional problems while enhancing patient outcomes, making them an essential component of clinical nutritional therapies.

In the realm of cardiovascular disorders, high-density lipoprotein cholesterol (HDL-C) is a crucial biomarker that is intimately linked to the onset and advancement of numerous chronic illnesses (1). Numerous studies have documented that HDL-C exerts multidimensional protective effects in the pathogenesis of atherosclerotic cardiovascular disease through mechanisms such as reverse cholesterol transport, anti-inflammatory properties, and endothelial protection (2–5). Research has confirmed that targeted nutritional interventions can effectively increase HDL-C levels, thereby reducing the risk of cardiovascular diseases. Dietary interventions high in fiber, omega-3 fatty acids, and antioxidants, for instance, have been shown to lower inflammation and have a favorable impact on HDL-C levels (6), highlighting the significance of nutrition in the management of chronic diseases.

Literature reviews reveal that most studies on nutritional screening, assessment, and intervention focus on their correlation with specific diseases (7–10), and few investigate their relationship with HDL-C levels. This study aims to fill this gap. Therefore, this study focuses on the nutritional status and HDL-C levels of newly hospitalized (non-emergency) patients in Jiangsu Province. The objective is to analyze the relationship between nutritional risk screening, assessment, and intervention and HDL-C levels in newly hospitalized patients, providing scientific evidence for clinical nutritional intervention and chronic disease management.

## 2 Methods

### 2.1 Subject of the study

**Figure 1:**
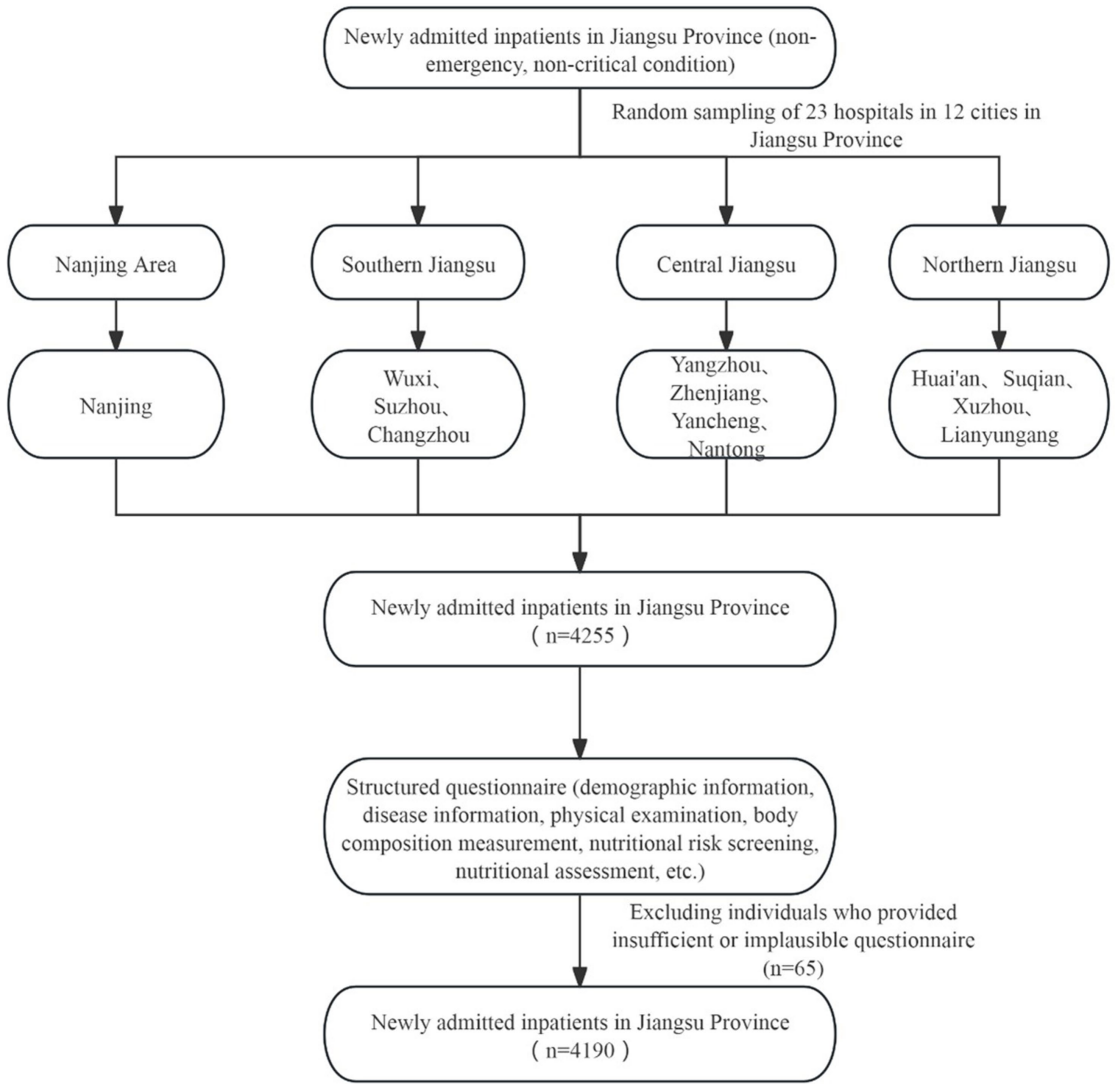
Flowchart of newly admitted inpatients through the study.

This study collected data from the “China Nutrition Fundamental Data Construction Project” in Jiangsu Province. The study population consisted of newly admitted, non-emergency patients. Inclusion criteria: newly admitted (non-emergency, non-critical condition) patients with one of the following seven system diseases—digestive, respiratory, cardiovascular and endocrine, oncology, neurological, and urinary systems; age ≥18 years; admission within 24–48 h. Exclusion criteria: pediatric and critically ill patients; individuals with psychiatric disorders or memory impairments who could not answer questions accurately; those lacking the capacity to perform behaviors; and other conditions deemed inappropriate for inclusion by the researchers (Figure 1).

### 2.2 Sampling methods

This multicenter cross-sectional study utilized data from newly hospitalized patients in Jiangsu Province, sourced from the region’s National Nutritional Basic Database Project. The research employed a multi-stage stratified cluster sampling method based on China’s administrative divisions. Initially, researchers identified all 31 provinces within mainland China, including autonomous areas and municipalities. In the second phase, they appointed one prominent hospital in each province (district and city) as the provincial lead hospital. They randomly picked between 2 and 25 secondary or tertiary hospitals in each province, district, and city. Jiangsu Provincial People’s Hospital, the leading hospital in Jiangsu Province, randomly selected 23 secondary or tertiary hospitals from 12 cities in the province between October 2020 and June 2021. During the third stage, a survey was administered to patients with seven systemic diseases—digestive, respiratory, cardiovascular, endocrine, neoplastic, neurological, and urological—by using a fixed continuous convenience sampling method in each randomly selected hospital in Jiangsu Province, continuing until a total of 200 observed cases was attained in each facility. The study received approval from the Ethics Committee of Peking Union Medical College Hospital (Approval Number: ZS-2614).

### 2.3 Blood sample collection and testing

We collected 3 mL of fasting venous blood in the morning after admission. We allowed the blood to stand for 30 min, then centrifuged at 1000 g for 10 min. We separated the serum and plasma, and stored them in a low-temperature freezer at −80°C for batch testing later. A professional laboratory physician measured the serum HDL-C concentration using a homogeneous method.

### 2.4 Forms of investigation

This survey employed a dual reporting method using both paper and electronic questionnaires. The survey included patient demographic information, disease information, physical examination, body composition measurement, nutritional risk screening, and nutritional assessment. Initially, the paper questionnaire needed to be filled out. Within 1 week after the completion of each case survey, the electronic questionnaire was filled out on the National Nutrition Database platform based on the content of the paper questionnaire.

### 2.5 Organization and implementation of the survey

This survey was jointly led by the National Health Commission and the Health Commission Hospital Management Research Institute, with a leadership group established for oversight. The Health Commission Hospital Management Research Institute managed the day-to-day activities. A project expert group and an execution group (including quality control and data management teams) of experts in relevant fields nationwide were organized, with a project office set up at Peking Union Medical College Hospital. This office was responsible for specific project organization, communication and coordination, technical guidance and quality control, data compilation, and project summarization. A central office was established in Jiangsu Province to manage 23 hospitals across 12 cities, with each hospital tasked to complete 185 cases. The Jiangsu central office was responsible for forming local expert groups, leadership and execution teams (including quality control and data management teams), and organizing survey teams at each investigation site. This office oversaw the provincial survey’s organization, implementation, quality control, data entry, and reporting. The project execution group was responsible for detailed project implementation, cooperating with the national level to complete on-site surveys, data verification, and reporting.

### 2.6 Organization of survey information

The project uniformly assigned survey center numbers, ID numbers, and data entry procedures. Each investigation site inputs the collected data into the computer using the program uniformly compiled by the project after verifying that the collected data were correct. Then, they established a database and reported it to the general project office. The general project office further verified and cleaned the data. Once data cleaning was completed, statistical analysis was performed. All data entry and reporting must be completed within 3 months of the end of the on-site survey. The original survey forms were retained at each provincial center for reference.

### 2.7 Statistical analysis

Statistical analysis was conducted using SPSS 25.0. The mean ± standard deviation was employed to represent normally distributed continuous data, and the independent samples t-test was utilized to compare groups. Non-parametric tests contrasted groups and indicated non-normally distributed continuous data as the median (interquartile range). Frequencies (percentages) were employed to delineate the characteristics of newly admitted patients with and without nutritional risk; the chi-square test was used for group comparison. Frequencies (percentages) were employed to delineate the attributes of HDL-C levels (mmol/L) concerning dietary risk assessment, evaluation, and intervention. The chi-square test was employed to compare the groups. Linear and binary logistic regression analyses compared HDL-C values with nutritional risk screening, evaluation, and intervention. *p*-values below 0.05 were considered statistically significant.

Definition of indicators: Referencing the “*Chinese Guidelines for the Management of Dyslipidemia*” (11), HDL-C < 1 mmol/L was classified as low HDL-C dyslipidemia. Nutritional risk screening: The Nutritional Risk Screening 2002 (NRS2002) score collected within 24 h of hospital admission assessed nutritional risk, with a score ≥ 3 indicating nutritional risk and < 3 indicating no nutritional risk (12). Nutritional assessment used the Global Leadership Initiative on Malnutrition (GLIM) criteria (13). Nutritional support encompassed the dietary interventions employed during a patient’s hospitalization, including oral meals, oral nutritional supplements, enteral nutrition through tube feeding, and parenteral nutrition. Each patient could get one or more forms of nutritional support (14).

Quality control: The Jiangsu Provincial Center had established a provincial quality control working group to oversee the entire quality control process of the provincial survey, including sampling, questionnaire surveys, physical examinations, laboratory tests, and data management, according to the project quality control standards and methods. Each district/county survey site appointed a dedicated person responsible for quality control at each stage. The survey work plan, measurement tools, and quality control methods at each stage were standardized; rigorous training and assessments ensured the implementation of quality control measures. External forces were introduced to supervise and evaluate the project externally.

## 3 Results

### 3.1 Prevalence of low HDL-C in different population characteristics

Table 1 illustrates the levels of HDL-C in newly hospitalized patients and the nutritional risk characteristics of the general population. 4,190 patients were included, with a prevalence of low HDL-C of 30.7%. Among them, 2,377 were male (56.7%) and 1813 were female (43.3%). The prevalence of low HDL-C was significantly higher in males (35.9%) compared to females (23.9%). In different age groups, the prevalence rates were as follows: 22.0% in the 18–24 years group, 41.0% in the 25–29 years group, 32.5% in the 30–39 years group, 29.5% in the 40–49 years group, 29.8% in the 50–59 years group, 30.4% in the 60–69 years group, 31.0% in the 70–79 years group, 31.7% in the 80–89 years group, and 61.1% in the 90–99 years group. The prevalence of low HDL-C showed an “N”-shaped distribution, initially increasing, then decreasing, and finally increasing again with age.

**Table 1:** The levels of HDL-C (mmol/L) in newly hospitalized patients and the nutritional risk characteristics of the general population in 23 hospitals across 12 cities in Jiangsu Province.

Characteristics	N	HDL-C < 1	HDL-C ≥ 1	*χ^2^*	*p*	HDL concentration	*p*	N	No nutritional risk	Nutritional risk	*χ^2^*	*p*
		N(%)	N(%)			*P*_50_ (*P*_25_–*P*_75_)			N(%)	N(%)		
Age, years												
18–24	59	13(22.0%)	46(78.0%)	13.718	0.089	1.195(1.000–1.605)	0.247	62	53(85.5%)	9(14.5%)	375.614	**< 0.001**
25–29	83	34(41.0%)	49(59.0%)			1.070(0.830–1.410)		89	78(87.6%)	11(12.4%)		
30–39	255	83(32.5%)	172(67.5%)			1.090(0.890–1.419)		286	271(94.8%)	15(5.2%)		
40–49	432	128(29.5%)	304(70.5%)			1.110(0.880–1.420)		473	431(91.1%)	42(8.9%)		
50–59	1,038	309(29.8%)	729(70.2%)			1.130(0.880–1.447)		1,135	1,056(93.0%)	79(7.0%)		
60–69	1,091	362(30.4%)	829(69.6%)			1.130(0.880–1.450)		1,315	1,199(91.2%)	116(8.8%)		
70–79	887	275(31.0%)	612(69.0%)			1.110 (0.870–1.460)		959	683(71.2%)	276(28.8%)		
80–89	227	72(31.7%)	155(68,3%)			1.090 (0.843–1.386)		249	165(66.3%)	84(33.7%)		
90–99	18	11(61.1%)	7(38.9%)			0.920(0.797–1.153)		22	12(54.5%)	10(45.5%)		
Age, years												
18–24	59	13(22.0%)	46(78.0%)	12.174	**0.007**	1.195(1.000–1.605)	**0.048**	62	53(85.5%)	9(14.5%)	18.375	**< 0.001**
25–29	83	34(41.0%)	49(59.0%)			1.070(0.830–1.410)		89	78(87.6%)	11(12.4%)		
30–89	4,030	1,229(30.5%)	2,801(69.5%)			1.120(0.880–1.440)		4,417	3,805(86.1%)	612(13.9%)		
90–99	18	11(61.1%)	7(38.9%)			0.920(0.797–1.153)		22	12(54.5%)	10(45.5%)		
Sex												
Male	2,377	854(35.9%)	1,523(64.1%)	70.113	**<0.001**	1.060(0.840–1.380)	**<0.001**	2,640	2,252(85.3%)	388(14.7%)	2.604	0.107
Female	1813	433(23.9%)	1,380(76.1%)			1.200(0.940–1.510)		1950	1,696(87.0%)	254(13.0%)		
Nationality												
Han	4,177	1,282(30.7%)	2,895(69.3%)	0.368	0.544	1.120(0.870–1.440)	0.601	4,576	3,935(86.0%)	641(14.0%)	0.547	0.460
Others	13	5(38.5%)	8(61.5%)			1.115(0.825–1.338)		14	13(92.9%)	1(7.1%)		
Regional Distribution												
Nanjing Area	1,105	282(25.5%)	823(74.5%)	19.573	**<0.001**	1.160(0.920–1.480)	**0.003**	1,204	1,074(89.2%)	130(10.8%)	15.928	**0.001**
Southern Jiangsu	1,103	356(32.3%)	747(67.7%)			1.090(0.860–1.410)		1,196	1,003(83.9%)	193(16.1%)		
Central Jiangsu	1,084	348(32.1%)	736(67.9%)			1.101(0.860–1.410)		1,195	1,015(84.9%)	180(15.1%)		
Northern Jiangsu	898	301(33.5%)	597(66.5%)			1.110(0.840–1.470)		995	856(86.0%)	139(14.0%)		
City Distribution												
Nanjing	1,105	282(25.5%)	823(74.5%)	63.911	**<0.001**	1.160(0.920–1.480)	**<0.001**	1,204	1,074(89.2%)	130(10.8%)	68.19	**< 0.001**
Xuzhou	364	129(35.4%)	235(64.6%)			1.130(0.810–1.520)		404	354(87.6%)	50(12.4%)		
Lianyungang	175	54(30.9%)	121(69.1%)			1.100(0.870–1.390)		196	161(82.1%)	35(17.9%)		
Suqian	177	43(24.3%)	134(75.7%)			1.100(0.940–1.310)		201	173(86.1%)	28(13.9%)		
Yangzhou	357	139(38.9%)	218(61.1%)			1.050(0.730–1.461)		398	336(84.4%)	62(15.6%)		
Zhenjiang	359	103(28.7%)	256(71.3%)			1.110(0.900–1.374)		398	337(84.7%)	61(15.3%)		
Yancheng	181	48(26.5%)	143(73.5%)			1.125(0.913–1.340)		200	173(86.5%)	27(13.5%)		
Wuxi	176	74(42.0%)	102(58.0%)			1.005(0.828–1.243)		198	143(72.2%)	55(27.8%)		
Suzhou	382	137(35.9%)	245(64.1%)			1.175(0.710–1.728)		398	318(79.9%)	80(20.1%)		
Changzhou	545	145(26.6%)	400(73.4%)			1.110(0.840–1.530)		600	542(90.3%)	58(9.7%)		
Nantong	187	58(31.0%)	129(69.0%)			1.180(0.840–1.530)		199	169(84.9%)	30(15.1%)		
Huai’an	182	75(41.2%)	107(58.8%)			1.102(0.304–1.944)		194	168(86.6%)	26(13.4%)		
Education level												
No schooling	560	148(26.4%)	412(73.6%)	5.766	0.124	1.160(0.920–1.440)	0.382	611	474(77.6%)	137(22.4%)	54.067	**< 0.001**
Primary to High School (Technical secondary school)	3,103	975(31.4%)	2,128(68.6%)			1.110(0.870–1.440)		3,391	2,933(86.5%)	458(13.5%)		
Bachelor	506	160(31.6%)	346(68.4%)			1.090(0.870–1.490)		559	513(91.8%)	46(8.2%)		
Master’s and Above	21	6(28.6%)	15(71.4%)			1.135(0.908–1.323)		24	23(95.8%)	1(4.2%)		
Season												
Spring	130	47(36.2%)	83(63.8%)	7.347	0.062	1.260(0.561–1.740)	0.349	139	123(88.5%)	16(11.5%)	35.362	**< 0.001**
Summer	30	13(43.3%)	17(56.7%)			1.000(0.559–1.741)		33	24(72.7%)	9(27.3%)		
Autumn	1872	544(29.1%)	1,328(70.9%)			1.130(0.890–1.440)		2068	1842(89.1%)	226(10.9%)		
Winter	2,158	683(31.6%)	1,475(68.4%)			1.110(0.860–1.420)		2,350	1959(83.4%)	391(16.6%)		
Diagnosis of Infectious and Parasitic Diseases												
No	4,145	1,267(30.6%)	2,878(69.4%)	4.029	**0.045**	1.120(0.990–1.440)	**0.042**	4,539	3,903(86.0%)	636(14.0%)	0.212	0.645
Yes	45	20(44.4%)	25(55.6%)			0.980(0.690–1.350)		51	45(88.2%)	6(11.8%)		
Diagnosis of Tumor												
No	3,130	1,007(32.2%)	2,123(67.8%)	12.334	**<0.001**	1.090(0.865–1.400)	**<0.001**	3,457	3,058(88.5%)	399(11.5%)	69.598	**< 0.001**
Yes	1,060	280(26.4%)	780(73.6%)			1.210(0.910–1.610)		1,133	890(78.6%)	243(21.4%)		
Diagnosis of Blood and Hematopoietic System Diseases												
No	4,077	1,256(30.8%)	2,821(69.2%)	0.588	0.443	1.120(0.870–1.440)	0.808	4,464	3,836(85.9%)	628(14.1%)	0.891	0.345
Yes	113	31(27.4%)	82(72.6%)			1.090(0.904–1.413)		126	112(88.9%)	14(11.1%)		
Diagnosis of Endocrine, Nutritional, and Metabolic Diseases												
No	3,124	920(29.4%)	2,204(70.6%)	9.256	**0.002**	1.140(0.880–1.470)	**<0.001**	3,386	2,877(85.0%)	509(15.0%)	11.730	**0.001**
Yes	1,066	367(34.4%)	699(65.6%)			1.060(0.860–1.364)		1,204	1,071(89.0%)	133(11.0%)		
Diagnosis of Nervous System Diseases												
No	3,577	1,082(30.2%)	2,495(69.8%)	2.507	0.113	1.130(0.874–1.460)	**0.003**	3,906	3,410(87.3%)	496(12.7%)	36.173	**< 0.001**
Yes	613	205(33.4%)	408(66.6%)			1.07(0.870–1.366)		684	538(78.7%)	146(21.3%)		
Diagnosis of Circulatory System Diseases												
No	2,595	765(29.5%)	1830(70.5%)	4.895	**0.027**	1.150(0.880–1.520)	**<0.001**	2,802	2,376(84.8%)	426(15.2%)	8.848	**0.003**
Yes	1,595	522(32.7%)	1,073(67.3%)			1.070(0.870–1.340)		1788	1,572(87.9%)	216(12.1%)		
Diagnosis of Respiratory System Diseases												
No	3,488	1,052(30.2%)	2,436(69.8%)	3.018	0.082	1.120(0.880–1.440)	0.210	3,837	3,327(86.7%)	510(13.3%)	9.399	**0.002**
Yes	702	235(33.5%)	467(66.5%)			1.110(0.813–1.470)		753	621(82.5%)	132(17.5%)		
Diagnosis of Digestive System Diseases												
No	3,395	1,031(30.4%)	2,364(69.6%)	1.017	0.313	1.120(0.880–1.450)	0.312	3,730	3,223(86.4%)	507(13.6%)	2.574	0.109
Yes	795	256(32.2%)	539(67.8%)			1.110(0.850–1.410)		860	725(84.3%)	135(15.7%)		
Diagnosis of Genitourinary System Diseases												
No	3,517	1,047(29.8%)	2,470(70.2%)	9.214	**0.002**	1.120(0.880–1.430)	0.526	3,868	3,304(85.4%)	564(14.6%)	7.218	**0.007**
Yes	673	240(35.7%)	433(64.3%)			1.110(0.820–1.500)		722	644(89.2%)	78(10.8%)		
Number of Diagnosed Diseases												
Single Disease	2,192	618(28.2%)	1,574(71.8%)	13.745	**<0.001**	1.120(0.896–1.470)	**<0.001**	2,393	2,108(88.1%)	285(11.9%)	17.93	**< 0.001**
Multiple Diseases	1998	669(33.5%)	1,329(66.5%)			1.080(0.850–1.400)		2,197	1840(83.8%)	357(16.2%)		

The bold values are statistically significant, with *p*-values all less than 0.05. In addition, the numbers in bold use the same statistical method. Mann Whitney U Nonparametric Test or K Independent Samples Median Nonparametric Test was used to describe median differences by continuous variables and the chi-square test was used to examine differences in categorical variables.

After classifying and statistically analyzing the data based on age, sex, regional distribution, urban distribution, and the presence of various diseases, the results indicated significant differences in the prevalence of low HDL-C associated with these factors. Higher prevalence was observed in patients aged 90–99, males, those from Northern Jiangsu, and patients from Wuxi. Patients with infectious and communicable diseases, endocrine, nutritional, and metabolic diseases, circulatory system diseases, and genitourinary system diseases were more likely to have low HDL-C. Conversely, patients diagnosed with tumors were less likely to have low HDL-C. The incidence of low HDL-C was elevated in patients with numerous comorbidities. All findings were statistically significant (*p* < 0.05).

### 3.2 Nutritional risk profile analysis of different characterized populations

Researchers enrolled 4,590 patients in the nutritional risk screening study, including 2,640 males and 1,950 females. They analyzed the nutritional risk profiles of newly hospitalized patients and found significant variations in the prevalence of nutritional risk across different demographic and clinical characteristics. For sex, the nutritional risk percentages for males and females were 14.7 and 13%, respectively, with no statistically significant difference (*p* > 0.05). With age grouping, the proportion of patients at nutritional risk increased significantly, especially in patients over 70, where the nutritional risk ranged from 28.8 to 45.5%. The higher the education level, the lower the proportion of patients at nutritional risk, with the highest risk in patients with no formal education (22.4%) and the lowest in those with a master’s degree or higher (4.2%). Regionally, the lowest nutritional risk proportion was found in Nanjing (10.8%), while the highest was in southern Jiangsu (16.1%). Among cities, Wuxi and Suzhou had the highest nutritional risk proportions (27.8 and 20.1%, respectively), while Changzhou and Nanjing had the lowest (9.7 and 10.8%, respectively). Seasonally, patients admitted in autumn had the lowest nutritional risk proportion (10.9%), while those admitted in winter had the highest (16.6%). Regarding the type of diagnosis, patients with tumors, neurological diseases, and respiratory diseases had significantly higher nutritional risk proportions than other disease types (all *P*s<0.05).

According to Table 2, only 2.0% of patients with normal nutritional status exhibited nutritional risks, compared to 94.9 to 100% among patients experiencing significant weight loss. Similarly, disease severity scores indicated that patients with severe diseases had substantially higher nutritional risks than healthy individuals (all *P*s<0.001).

**Table 2:** The relationship between HDL-C levels (mmol/L) and nutritional risk screening, assessment, and intervention in newly hospitalized patients in 23 hospitals across 12 cities in Jiangsu Province.

Characteristics	N	HDL-C < 1	HDL-C ≥ 1	*χ^2^*	*p*	HDL concentration	*p*	N	No nutritional risk	Nutritional risk	*χ^2^*	*p*
		N(%)	N(%)			*P*_50_ (*P*_25_*–P*_75_)			N(%)	N(%)		
Nutritional Status Score												
0	3,337	999(29.9%)	2,338(70.1%)	8.163	**0.043**	1.120(0.880–1.430)	0.222	3,668	3,594(98.0%)	74(2.0%)	2857.21	**< 0.001**
1	509	164(32.2%)	345(67.8%)			1.132(0.840–1.503)		544	344(63.2%)	200(36.8%)		
2	178	52(32.6%)	120(67.4%)			1.120(0.860–1.560)		195	10(5.1%)	185(94.9%)		
3	166	66(39.8%)	100(60.2%)			1.030(0.750–1.380)		183	0	183(100%)		
Disease severity score												
0	1,056	328(31.1%)	728(68.9%)	7.609	0.055	1.120(0.874–1.430)	0.085	1,158	1,145(98.9%)	13(1.1%)	748.376	**< 0.001**
1	2,846	851(29.9%)	1995(70.1%)			1.120(0.880–1.450)		3,115	2,677(85.9%)	438(14.1%)		
2	270	100(37.0%)	170(63.0%)			1.070(0.790–1.430)		299	126(42.1%)	173(57.9%)		
3	18	8(44.4%)	10(55.6%)			1.065(0.638–1.153)		18	0	18(100%)		
Age Score												
0 points: <70 years old	3,058	929(30.4%)	2,129(69.6%)	0.603	0.438	1.120(0.870–1.440)	0.608	3,361	3,088(91.9%)	273(8.1%)	358.824	**< 0.001**
1 point: ≥70 years old	1,132	358(31.6%)	774(68.4%)			1.110(0.860–1.430)		1,229	860(70.0%)	369(30.0%)		
NRS2002 Score Classification												
No nutritional risk (total score < 3)	3,597	1,082(30.1%)	2,515(69.9%)	4.821	**0.028**	1.120(0.880–1.430)	0.506	4,226	3,832(90.7%)	394(9.3%)	963.415	**< 0.001**
Nutritional risk (total score ≥ 3)	593	205(34.6%)	388(65.4%)			1.100(0.830–1.505)		364	116(31.9%)	248(68.1%)		
Weight loss >5% over 6 months												
Yes	549	183(33.3%)	366(66.7%)	2.022	0.155	1.090(0.840–1.489)	0.469	602	254(42.2%)	348(57.8%)	1105.59	**< 0.001**
No	3,640	1,104(30.3%)	2,536(69.7%)			1.120(0.880–1.430)		3,987	3,693(92.6%)	294(7.4%)		
Weight loss >10% over 6 months												
Yes	128	45(35.2%)	83(64.8%)	1.219	0.270	1.130(0.855–1.435)	0.906	137	55(40.1%)	82(59.9%)	246.866	**< 0.001**
No	4,061	1,242(30.6%)	2,819(69.4%)			1.120(0.870–1.440)		4,452	3,892(87.4%)	560(12.6%)		
Less than the recommended energy intake for more than 2 weeks												
Yes	552	172(31.2%)	380(68.8%)	0.057	0.812	1.110(0.860–1.458)	0.816	600	262(43.7%)	338(56.3%)	1028.513	**< 0.001**
No	3,637	1,115(30.7%)	2,522(69.3%)			1.120(0.880–1.440)		3,989	3,685(92.4%)	304(7.6%)		
Less than the recommended energy intake for more than 1 week												
Yes	160	54(33.8%)	106(66.3%)	0.716	0.397	1.080(0.840–1.420)	0.497	173	33(19.1%)	140(80.9%)	669.382	**< 0.001**
No	4,029	1,233(30.6%)	2,796(69.4%)			1.120(0.880–1.440)		4,416	3,914(88.6%)	502(11.4%)		
Dysphagia												
Yes	112	30(26.8%)	82(73.2%)	0.838	0.360	1.142(0.880–1.415)	0.804	117	55(47.0%)	62(53.0%)	151.774	**< 0.001**
No	4,077	1,257(30.8%)	2,820(69.2%)			1.120(0.870–1.440)		4,472	3,892(87.0%)	580(13.0%)		
Nausea and Vomiting												
Yes	328	99(30.2%)	229(69.8%)	0.049	0.825	1.115(0.870–1.428)	0.521	360	207(57.5%)	153(42.5%)	263.883	**< 0.001**
No	3,861	1,188(30.8%)	2,673(69.2%)			1.120(0.870–1.440)		4,229	3,740(88.4%)	489(11.6%)		
Diarrhea												
Yes	176	53(30.1%)	123(69.9%)	0.032	0.858	1.110(0.880–1.483)	0.870	198	132(66.7%)	66(33.3%)	64.345	**< 0.001**
No	4,013	1,234(30.8%)	2,779(69.2%)			1.120(0.870–1.440)		4,391	3,815(86.9%)	576(13.1%)		
Constipation												
Yes	248	95(38.3%)	153(61.7%)	7.122	**0.008**	1.050(0.840–1.388)	0.051	271	184(67.9%)	87(32.1%)	78.53	**< 0.001**
No	3,941	1,192(30.2%)	2,749(69.8%)			1.120(0.880–1.440)		4,318	3,763(87.1%)	555(12.9%)		
Abdominal Pain												
Yes	264	81(30.7%)	183(69.3%)	0	0.988	1.152(0.880–1.490)	0.440	287	189(65.9%)	98(34.1%)	103.368	**< 0.001**
No	3,925	1,206(30.7%)	2,719(69.3%)			1.110(0.870–1.440)		4,302	3,758(87.4%)	544(12.6%)		
Pancreatic Insufficiency												
Yes	40	14(35.0%)	26(65.0%)	0.347	0.556	1.140(0.660–1.502)	0.764	41	30(73.2%)	11(36.8%)	5.668	**0.017**
No	4,149	1,273(30.7%)	2,876(69.3%)			1.120(0.870–1.440)		4,548	3,917(86.1%)	631(13.9%)		
Esophageal Stricture												
Yes	47	7(14.9%)	40(85.1%)	5.596	**0.018**	1.205(1.028–1.473)	0.130	50	24(48.0%)	26(52.0%)	60.696	**< 0.001**
No	4,142	1,280(30.9%)	2,862(69.1%)			1.120(0.870–1.440)		4,539	3,923(86.4%)	616(13.6%)		
Intestinal Obstruction												
Yes	16	7(43.8%)	9(56.2%)	1.281	0.258	1.115(0.657–1.338)	0.601	16	8(50.0%)	8(50.0%)	17.303	**< 0.001**
No	4,173	1,280(30.7%)	2,893(69.3%)			1.120(0.870–1.440)		4,573	3,939(86.1%)	634(13.9%)		
Large-volume Gastrostomy												
Yes	12	2(16.7%)	10(83.3%)	1.117	0.291	1.100(0.940–1.290)	0.899	15	10(66.7%)	5(33.3%)	4.68	**0.031**
No	4,177	1,285(30.8%)	2,892(69.2%)			1.120(0.870–1.440)		4,574	3,937(86.1%)	637(13.9%)		
Reduced Food Intake or Absorption												
Yes	1,194	383(32.1%)	811(67.9%)	1.438	0.230	1.110(0.868–1.430)	0.310	1,306	848(64.9%)	458(35.1%)	674.096	**< 0.001**
No	2,995	904(30.2%)	2091(69.8%)			1.120(0.880–1.440)		3,283	3,099(94.4%)	184(5.6%)		
Severe Infection												
Yes	106	55(51.9%)	51(48.1%)	22.885	**<0.001**	0.940(0.505–1.260)	**< 0.001**	115	79(68.7%)	36(31.3%)	29.388	**< 0.001**
No	4,083	1,232(30.2%)	2,851(69.8%)			1.120(0.880–1.445)		4,474	3,868(86.5%)	606(13.5%)		
Trauma												
Yes	16	6(37.5%)	10(62.5%)	0.347	0.556	1.159(0.600–1.435)	0.722	16	6(37.5%)	10(62.5%)	31.4	**< 0.001**
No	4,173	1,281(30.7%)	2,892(69.3%)			1.120(0.870–1.440)		4,573	3,941(86.2%)	632(13.8%)		
Closed Head Injury												
Yes	46	4(8.7%)	42(91.3%)	10.603	**0.001**	1.275(1.073–1.558)	**0.007**	48	29(60.4%)	19(39.6%)	26.406	**< 0.001**
No	4,143	1,283(31.0%)	2,860(69.0%)			1.110(0.870–1.440)		4,541	3,918(86.3%)	623(13.7%)		
Heart Failure												
Yes	108	42(38.9%)	66(61.1%)	3.473	0.062	1.025(0.840–1.170)	**< 0.001**	126	97(77.0%)	29(23.0%)	8.772	**0.003**
No	4,081	1,245(30.5%)	2,836(69.5%)			1.120(0.880–1.450)		4,463	3,850(86.3%)	613(13.7%)		
Chronic Obstructive Pulmonary Disease (COPD)												
Yes	106	35(33.0%)	71(67.0%)	0.269	0.604	1.080(0.843–1.410)	0.402	120	85(70.8%)	35(29.2%)	23.587	**<0.001**
No	4,083	1,252(30.7%)	2,831(69.3%)			1.120(0.871–1.440)		4,469	3,862(86.4%)	607(13.6%)		
Chronic Kidney Disease												
Yes	263	105(39.9%)	158(60.1%)	11.161	**0.001**	1.030(0.788–1.343)	**0.003**	290	231(79.7%)	59(20.3%)	10.389	**0.001**
No	3,926	1,182(30.1%)	2,744(69.9%)			1.120(0.880–1.450)		4,299	3,716(86.4%)	583(13.6%)		
Malignant Tumor												
Yes	1,081	288(26.6%)	793(73.4%)	11.403	**0.001**	1.190(0.910–1.590)	**<0.001**	1,157	905(78.2%)	252(21.8%)	78.031	**< 0.001**
No	3,108	999(32.1%)	2,109(67.9%)			1.090(0.860–1.400)		3,432	3,042(88.6%)	390(11.4%)		
Fever												
Yes	79	45(57.0%)	34(43.0%)	26.045	**<0.001**	0.850(0.480–1.260)	**<0.001**	83	47(56.6%)	36(43.4%)	60.652	**< 0.001**
No	4,110	1,242(30.2%)	2,868(69.8%)			1.120(0.880–1.440)		4,506	3,900(86.6%)	606(13.4%)		
High CRP												
Yes	546	235(43.0%)	311(57.0%)	38.021	**<0.001**	1.010(0.715–1.395)	**<0.001**	593	415(70.0%)	178(30.0%)	161.363	**< 0.001**
No	2,865	849(29.6%)	2016(70.4%)			1.120(0.889–1.420)		3,155	2,823(89.5%)	332(10.5%)		
Hypoalbuminemia												
Yes	243	128(52.7%)	115(47.3%)	58.357	**<0.001**	0.910(0.521–1.310)	**<0.001**	256	150(58.6%)	106(41.4%)	169.297	**< 0.001**
No	3,945	1,159(29.4%)	2,786(70.6%)			1.130(0.890–1.440)		4,332	3,796(87.6%)	536(12.4%)		
Presence of disease burden or inflammatory condition												
Yes	2,341	728(31.1%)	1,613(68.9%)	0.336	0.562	1.120(0.870–1.460)	0.767	2,548	2044(80.2%)	504(19.8%)	159.472	**< 0.001**
No	1847	559(30.3%)	1,288(69.7%)			1.118(0.880–1.420)		2040	1902(93.2%)	138(6.8%)		
Nutritional Intervention Methods												
No Intervention	2049	601(29.3%)	1,448(70.7%)	11.231	**0.011**	1.140(0.879–1.480)	**0.004**	2,230	1939(87.0%)	291(13.0%)	93.316	**< 0.001**
Enteral Nutrition	2017	634(31.4%)	1,383(68.6%)			1.100(0.880–1.410)		2,227	1932(86.8%)	295(13.2%)		
Parenteral Nutrition	74	34(45.9%)	40(54.1%)			1.014(0.650–1.337)		76	41(53.9%)	35(46.1%)		
Combined	49	18(36.7%)	31(63.3%)			1.075(0.753–1.358)		56	35(62.5%)	21(37.5%)		

Nutritional Status Score Score 0: Normal nutritional status; Score 1: Weight loss >5% in 3 months or Food intake 50–75% of normal requirement in preceding week; Score 2: Weight loss >5% in 2 months or Food intake 25–50% of normal requirement in preceding week; Score 3: Weight loss >5% in 1 month (≈ 15% in 3 months) or BMI I < 18.5 kg/m^2^ or Food intake 0–25% of normal requirement in preceding week. Disease Severity Score Score 0: Normal nutritional requirements; Score 1 (weakened, but not bedridden): Chronically ill patients, in particular with acute complications, such as cirrhosis, COPD; Score 2 (confined to bed): Major abdominal surgery, stroke, severe infection; Score 3 (ventilated, inotropic drugs): Severe head injuries, bone marrow transplantation, intensive care patients with APACHE >10. The bold values are statistically significant, with p-values all less than 0.05. In addition, the numbers in bold use the same statistical method. Mann Whitney U Nonparametric Test or K Independent Samples Median Nonparametric Test was used to describe median differences by continuous variables and the chi-square test was used to examine differences in categorical variables.

Among nutritional assessment indicators, patients with reduced food intake or absorption had a nutritional risk proportion of 35.1%, significantly higher than the 5.6% seen in patients with regular intake. Patients with gastrointestinal symptoms that affect food intake or absorption—such as dysphagia (53%), nausea and vomiting (42.5%), diarrhea (33.3%), constipation (32.1%), abdominal pain (34.1%), pancreatic insufficiency (36.8%), esophageal stricture (52%), intestinal obstruction (50%), and large-output gastrointestinal stoma (33.3%)—had significantly higher nutritional risk proportions compared to those without such symptoms: dysphagia (13%), nausea and vomiting (11.6%), diarrhea (13.1%), constipation (12.9%), abdominal pain (12.6%), pancreatic insufficiency (13.9%), esophageal stricture (13.6%), intestinal obstruction (13.9%), and large-output gastrointestinal stoma (14%). Patients with other disease burdens or inflammation statuses, excluding chronic liver disease, also had significantly higher nutritional risk proportions than those without related medical history (all *P*s <0.05).

Regarding nutritional intervention approaches, the nutritional risk was reduced in patients who did not receive nutritional intervention and those who received enteral nutrition, at 13.0 and 13.2%, respectively. In contrast, the risk significantly increased in patients who received parenteral nutrition and combined interventions at 46.1 and 37.5%, respectively (all *P*s <0.05).

### 3.3 New inpatients with low HDL-C levels: chi-square test on nutritional risk screening, assessment, and intervention

#### 3.3.1 Nutritional risk screening

Table 2 demonstrates a significant variation in the probability of low HDL-C levels among individuals with differing dietary situations. The incidence of low HDL-C levels among patients with nutritional status scores of 0, 1, 2, and 3 points is 29.9, 32.2, 32.6, and 39.8%, respectively. Patients with elevated nutritional status scores exhibit a markedly higher frequency of low HDL-C values than those with normal nutritional status. Patients without nutritional risk exhibit a low HDL-C level prevalence of 30.1%, but those with nutritional risk (NRS2002 score ≥ 3) demonstrate a greater prevalence of 34.6%, with both comparisons yielding *p* < 0.05.

#### 3.3.2 Nutritional assessment

Regarding nutritional assessment, patients with reduced food intake or absorption exhibit different prevalence rates of low HDL-C levels. Patients with esophageal stricture have a significantly lower prevalence of low HDL-C levels at 14.9% compared to those without. Among patients with specific diseases or inflammatory conditions, those with a history of closed brain injury and malignant tumors have lower prevalence rates of low HDL-C levels at 8.7 and 26.6%, respectively. Conversely, patients with a history of severe infection, chronic kidney disease(CKD), fever, high CRP, and hypoalbuminemia have higher prevalence rates of low HDL-C levels at 51.9, 39.9, 57, 43, and 52.7%, respectively, all *P*s<0.05.

#### 3.3.3 Nutritional intervention

Nutritional intervention methods greatly influence the prevalence of low HDL-C levels. Patients lacking dietary supplementation exhibit a reduced prevalence of poor HDL-C values at 29.3%. In contrast, those who received parenteral nutrition and combined nutritional intervention have relatively higher prevalence rates of 45.9 and 36.7%, respectively, *p* < 0.05.

### 3.4 Odds ratios of low HDL-C levels in new hospitalized patients related to nutritional risk screening, assessment, and intervention

#### 3.4.1 Nutritional risk screening

**Figure 2:**
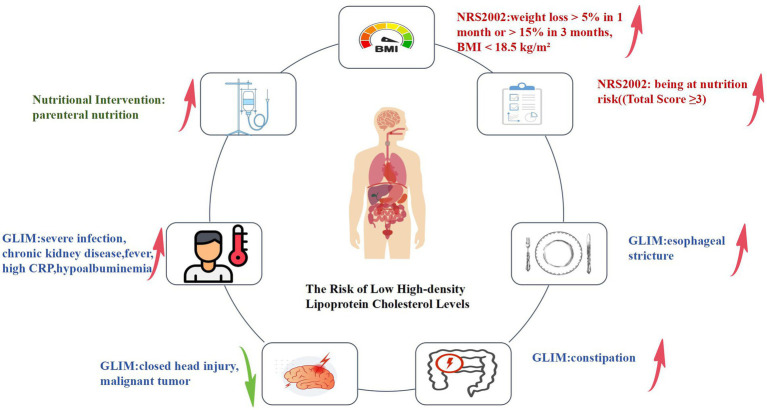
Logistic regression analysis of nutritional risk screening, nutritional assessment, nutritional intervention, and the risk of low HDL-C levels. In terms of nutritional risk screening(parts marked in red in the figure), those with “weight loss > 5% in 1 month or > 15% in 3 months, BMI < 18.5 kg/m^2^” and those at nutritional risk (total score ≥ 3) are associated with an increased risk of low HDL-C levels in these patients. In nutritional assessment indicators(parts marked in blue in the figure), among patients with reduced food intake or absorption, the presence of constipation and esophageal stricture is associated with an increased risk of low HDL-C levels. For patients with disease burdens or in inflammatory states, the presence of severe infection, chronic kidney disease, fever, high CRP, and hypoalbuminemia is associated with an increased risk of low HDL-C levels. Conversely, the presence of closed head injury and malignant tumors is associated with a decreased risk of low HDL-C levels in these patients. Regarding nutritional intervention indicators(parts marked in green in the figure), the use of parenteral nutrition is associated with an increased risk of low HDL-C levels in these patients.

As shown in Table 3 and Figure 2, compared to patients with normal nutritional status, those with “weight loss > 5% in 1 month or > 15% in 3 months, BMI < 18.5 kg/m^2^” and those at nutritional risk (total score ≥ 3) had a 1.545-fold and 1.228-fold increased risk of developing low HDL-C levels, respectively. After adjusting for confounders including age, sex, education level, region, diagnosis of infectious and parasitic diseases, neoplasms, hematological diseases, endocrine, nutritional and metabolic diseases, circulatory diseases, genitourinary diseases, and the number of diagnosed conditions, the results remained significant (all *P*s < 0.05).

**Table 3:** The unadjusted and adjusted odds ratios (95% CIs) and regression coefficients (95% CIs) for HDL-C levels and nutritional risk screening, assessment, and intervention in newly hospitalized patients.

Characteristics	N	OR (95%CI)	*p*	Adjusted OR (95%CI)	*p*	β (95%CI)	*p*	Adjusted β (95%CI)	*p*
Nutritional Status Score									
1	509	1.113(0.911,1.359)	0.296	1.068(0.866,1.317)	0.539	0.031(−0.085,0.147)	0.599	0.031(−0.081,0.143)	0.584
2	178	1.131(0.820,1.561)	0.453	1.216(0.870,1.701)	0.252	0.024(−0.161,0.210)	0.798	−0.002(−0.180,0.177)	0.986
3	166	1.545(1.122,2.126)	**0.008**	1.526(1.094,2.128)	**0.013**	−0.089(−0.280,0.103)	0.363	−0.107(−0.291,0.076)	0.252
0	3,337	1(ref)							
Disease severity score									
1	2,846	0.947(0.812,1.103)	0.484	0.944(0.794,1.123)	0.517	0.076(−0.011,0.163)	0.088	0.062(−0.029,0.152)	0.182
2	270	1.306(0.987,1.726)	0.061	1.218(0.905, 1.640)	0.193	−0.046(−0.209,0.118)	0.584	−0.028(−0.189,0.132)	0.728
3	18	1.776(0.694,4.540)	0.231	1.590(0.605,4.183)	0.347	−0.188(−0.787,0.411)	0.538	−0.212(−0.782,0.357)	0.465
0	1,056	1(ref)							
Age Score									
1 point: ≥70 years old	1,132	1.060(0.915,1.228)	0.438	1.033(0.882,1.210)	0.684	0.024(−0.060,0.108)	0.574	0.059(−0.024,0.143)	0.162
0 points: <70 years old	3,058	1(ref)							
NRS2002 Score Classification									
Nutritional risk (total score ≥3)	3,597	1.228(1.022,1.476)	**0.028**	1.225(1.009,1.486)	**0.040**	0.022(−0.085,0.130)	0.685	0.016(−0.088,0.120)	0.761
No Nutritional risk (total score < 3)	593	1(ref)							
Weight loss >5% over 6 months									
Yes	549	1.149(0.949,1.390)	0.155	1.186(0.969, 1.453)	0.098	0.004(−0.107,0.114)	0.948	−0.017(−0.125,0.091)	0.761
No	3,640	1(ref)							
Weight loss >10% over 6 months									
Yes	128	1.231(0.851,1.780)	0.270	1.303(0.891, 1.907)	0.173	0.058(−0.161,0.277)	0.606	0.025(−0.184,0.234)	0.817
No	4,061	1(ref)							
Less than the recommended energy intake for more than 2 weeks									
Yes	552	1.024(0.844,1.242)	0.812	1.048(0.855, 1.285)	0.652	0.028(−0.082,0.139)	0.615	0.012(−0.095,0.120)	0.822
No	3,637	1(ref)							
Less than the recommended energy intake for more than 1 week									
Yes	160	1.155(0.827,1.614)	0.398	1.199(0.846,1.699)	0.308	−0.089(−0.285,0.107)	0.372	−0.110(-0.298,0.078)	0.252
No	4,029	1(ref)							
Dysphagia									
Yes	112	0.821(0.537,1.254)	0.361	0.851(0.5501.318)	0.471	−0.139(−0.376,0.097)	0.248	−0.163(-0.390,0.064)	0.159
No	4,077	1(ref)							
Nausea and Vomiting									
Yes	328	0.973(0.761,1.243)	0.825	1.096(0.849,1.415)	0.482	−0.156(−0.294,-0.017)	**0.027**	−0.177(-0.310,-0.044)	**0.009**
No	3,861	1(ref)							
Diarrhea									
Yes	176	0.970(0.698,1.348)	0.858	0.934 (0.662,1.317)	0.697	−0.010(−0.193,0.174)	0.918	0.014(-0.162,0.189)	0.880
No	4,013	1(ref)							
Constipation									
Yes	248	1.432(1.099,1.866)	**0.008**	1.391(1.059,1.826)	**0.018**	−0.006(−0.164,0.152)	0.943	−0.009(-0.160,0.142)	0.908
No	3,941	1(ref)							
Abdominal Pain									
Yes	264	0.998(0.762,1.308)	0.988	1.102(0.831, 1.461)	0.499	−0.001(−0.155,0.153)	0.988	0.006(-0.142,0.153)	0.942
No	3,925	1(ref)							
Pancreatic Insufficiency									
Yes	40	1.217(0.633,2.337)	0.556	0.855(0.421,1.736)	0.665	−1.465(−2.923,-0.008)	**0.049**	−0.026(-0.412,0.361)	0.897
No	4,149	1(ref)							
Esophageal Stricture									
Yes	47	0.391(0.175,0.876)	**0.022**	0.452(0.199, 1.027)	0.058	0.026(−0.333,0.385)	0.886	−0.031(-0.377,0.315)	0.861
No	4,142	1(ref)							
Intestinal Obstruction									
Yes	16	1.758(0.653,4.731)	0.264	2.035(0.737, 5.618)	0.170	0.026(−0.606,0.658)	0.935	0.021(-0.577,0.619)	0.945
No	4,173	1(ref)							
Large-volume Gastrostomy									
Yes	12	0.450(0.098,2.057)	0.303	0.389(0.085,1.792)	0.226	−0.214(−0.867,0.439)	0.520	−0.167(-0.786,0.451)	0.595
No	4,177	1(ref)							
Reduced Food Intake or Absorption									
Yes	1,194	1.092(0.945,1.262)	0.231	1.124(0.965, 1.309)	0.132	−0.048(−0.130,0.035)	0.256	−0.049(-0.129,0.032)	0.235
No	2,995	1(ref)							
Severe Infection									
Yes	106	2.496(1.695,3.674)	**<0.001**	2.284(1.525,3.420)	**<0.001**	−0.403(−0.641,-0.165)	**0.001**	−0.381(-0.610,-0.152)	**0.001**
No	4,083	1(ref)							
Trauma									
Yes	16	1.355(0.491,3.735)	0.558	1.639(0.571,4.701)	0.358	−0.236(−0.868,0.396)	0.465	−0.283(-0.902,0.335)	0.369
No	4,173	1(ref)							
Closed Head Injury									
Yes	46	0.212(0.076,0.593)	**0.003**	0.174(0.061,0.491)	**0.001**	0.168(−0.199,0.534)	0.370	0.200(-0.150,0.550)	0.262
No	4,143	1(ref)							
Heart Failure									
Yes	108	1.450(0.979,2.147)	0.064	1.296(0.854, 1.966)	0.223	−0.162(−0.390,0.066)	0.163	−0.118(-0.343,0.106)	0.300
No	4,081	1(ref)							
Chronic Obstructive Pulmonary Disease (COPD)									
Yes	106	1.115(0.740,1.680)	0.604	0.982(0.643, 1.499)	0.932	−0.042(−0.276,0.191)	0.723	−0.013(-0.237,0.211)	0.910
No	4,083	1(ref)							
Chronic Kidney Disease									
Yes	263	1.543(1.194,1.993)	**0.001**	1.179(0.868, 1.600)	0.292	0.003(−0.151,0.156)	0.974	0.018(-0.151,0.186)	0.837
No	3,926	1(ref)							
Malignant Tumor									
Yes	1,081	0.767(0.657,0.895)	**0.001**	0.982(0.719, 1.341)	0.910	0.085(−0.001,0.170)	0.053	0.005(-0.162,0.172)	0.952
No	3,108	1(ref)							
Fever									
Yes	79	3.056(1.948,4.795)	**<0.001**	2.820(1.773,4.485)	**<0.001**	−0.301(−0.580,-0.021)	**0.035**	−0.279(-0.545,-0.013)	**0.040**
No	4,110	1(ref)							
High CRP									
Yes	546	1.794(1.488,2.164)	**<0.001**	1.759(1.441, 2.147)	**<0.001**	−0.089(−0.203,0.025)	0.125	−0.068(-0.176,0.041)	0.222
No	2,865	1(ref)							
Hypoalbuminemia									
Yes	243	2.703(2.082,3.509)	**<0.001**	2.648(2.006, 3.494)	**<0.001**	−0.250(−0.411,-0.089)	**0.002**	−0.247(-0.402,-0.092)	**0.002**
No	3,945	1(ref)							
Presence of disease burden or inflammatory condition									
Yes	2,341	1.040(0.911,1.187)	0.562	1.100(0.947, 1.279)	0.213	−0.007(−0.082,0.068)	0.854	−0.011(-0.091,0.068)	0.780
No	1847	1(ref)							
Nutritional Intervention Methods									
No Intervention	2049	1(ref)							
Enteral Nutrition	2017	1.104(0.966,1.263)	0.145	1.101(0.943, 1.287)	0.223	−0.050(−0.126,0.025)	0.191	−0.017(-0.099,0.065)	0.691
Parenteral Nutrition	74	2.048(1.284,3.267)	**0.003**	1.803(1.115,2.916)	**0.016**	−0.290(−0.585,0.004)	0.053	−0.258(-0.542,0.026)	0.075
Combined	49	1.399(0.777,2.520)	0.264	1.617(0.883,2.964)	0.120	−0.215(−0.556,0.127)	0.218	−0.229(-0.555,0.097)	0.169

The adjusted indicators include age, sex, patient education level, regional distribution, diagnosis of infectious and parasitic diseases, diagnosis of tumors, diagnosis of diseases of the blood and blood-forming organs, diagnosis of endocrine, nutritional, and metabolic diseases, diagnosis of circulatory system diseases, diagnosis of genitourinary system diseases, and the number of diagnosed diseases, in addition to the unadjusted basis. The bold values are statistically significant, with p-values all less than 0.05. In addition, the bold values use the same statistical method. Linear regression was used to assess the linear relationship between HDL-C levels and nutritional risk screening, evaluation, and intervention. Binary logistic regression was used to evaluate the association of these factors with dichotomous outcomes related to HDL-C levels.

#### 3.4.2 Nutritional assessment

In nutritional assessment indicators, among patients exhibiting reduced food intake or absorption, the likelihood of low HDL-C levels was significantly elevated by a factor of 1.432 in those experiencing constipation. In contrast, its % was significantly reduced by 60.9% in those with esophageal stricture (all *P*s < 0.05). Following adjustment for confounding variables, the risk of low HDL-C levels remained significantly increased by a factor of 1.391 in patients with constipation (*p* < 0.05), while it was reduced by 54.8% in those with esophageal stricture; however, this reduction was not statistically significant (*p* > 0.05). For patients with disease burdens or in inflammatory states, the risk of low HDL-C was significantly increased by 2.496 times in those with severe infections, 1.543 times in those with chronic kidney disease, 3.056 times in those with fever, 1.794 times in those with high CRP, and 2.703 times in those with hypoalbuminemia. The risk was decreased by 23.3% in patients with malignant tumors and by 78.8% in those with closed brain injuries. After adjusting for confounders, the risk of low HDL-C remained significantly increased by 2.284 times in patients with severe infections, 2.820 times in those with fever, 1.759 times in those with high CRP, and 2.648 times in those with hypoalbuminemia (all *P*s< 0.05). The increased risk of low HDL-C in patients with chronic kidney disease became weaker, with an increase of 1.179 times (*p* > 0.05). The risk of low HDL-C was significantly decreased by 82.6% in patients with a history of closed head injury (*p* < 0.05). For patients with a history of malignant tumors, the reduction in the risk of low HDL-C was relatively small, with only a 1.8% decrease (*p* > 0.05).

#### 3.4.3 Nutritional intervention

Regarding nutritional intervention, compared to patients who did not receive any intervention, those receiving parenteral nutrition had a significantly increased risk of low HDL-C levels by 2.048-fold. After adjusting for confounders, the risk increased considerably by 1.803-fold (*p* < 0.05).

### 3.5 Serum HDL-C levels in new hospitalized patients: nutritional risk screening, assessment, and intervention using rank-sum test

#### 3.5.1 Nutritional risk screening

Table 2 shows that there were differences in HDL-C levels among different nutritional statuses. The HDL-C levels of patients with a poor nutritional status score of 3, a disease severity score of 3, an age of 70 years and older, and those with nutritional risks were the lowest between groups, with 1.03 mmol/L, 1.065 mmol/L, 1.11 mmol/L, and 1.1 mmol/L, respectively. The differences were not statistically significant (*p* > 0.05).

#### 3.5.2 Nutritional assessment

Regarding nutritional assessment, serum HDL-C levels were found to vary under different disease burdens or inflammatory states. Patients with severe infections (0.94 mmol/L), heart failure (1.025 mmol/L), chronic kidney disease (1.03 mmol/L), fever (0.85 mmol/L), high CRP levels (1.01 mmol/L), and hypoalbuminemia (0.91 mmol/L) had significantly lower HDL-C levels compared to those without these conditions. Conversely, patients with a history of malignant tumors (1.19 mmol/L) and closed head injuries (1.275 mmol/L) had higher HDL-C levels compared to those without such histories (1.09 mmol/L and 1.11 mmol/L, respectively), with differences being statistically significant (*p* < 0.05).

#### 3.5.3 Nutritional interventions

Regarding nutritional interventions, patients who did not receive any intervention had HDL-C levels of 1.14 mmol/L, higher than those receiving enteral nutrition support (1.1 mmol/L) and combined enteral-parenteral nutrition support (1.075 mmol/L). Patients receiving parenteral nutrition support had the lowest HDL-C levels at 1.014 mmol/L. These differences were all statistically significant (*p* < 0.05).

### 3.6 Regression coefficients (95% CI) of serum HDL-C levels in newly hospitalized patients with nutritional risk screening, assessment, and intervention

#### 3.6.1 Nutritional risk screening

Regarding nutritional risk screening, patients with a nutritional status score of 3 and disease severity scores of 2 and 3 did not show significant reductions in HDL-C levels. After adjusting for confounding factors, the results remained unchanged. Patients aged 70 and above and those at nutritional risk did not exhibit significant increases in HDL-C levels, even after adjustment. No statistically significant differences were observed among the indicators (all *P*s > 0.05).

#### 3.6.2 Nutritional assessment

For nutritional assessment, patients with decreased food intake or absorption, specifically those experiencing nausea, vomiting, or pancreatic insufficiency, had reduced HDL-C levels by 0.156 mmol/L and 1.465 mmol/L, respectively (*p* < 0.05). After adjusting for confounders, HDL-C levels decreased by 0.177 mmol/L for patients with nausea and vomiting (*p* < 0.05) and by 0.026 mmol/L for those with pancreatic insufficiency. However, the latter was not statistically significant (*p* > 0.05). Regarding specific disease burdens or inflammatory states, patients with severe infections, fever, and hypoalbuminemia showed reductions in HDL-C levels by 0.403 mmol/L, 0.301 mmol/L, and 0.250 mmol/L, respectively. After adjustment, these reductions were 0.381 mmol/L, 0.279 mmol/L, and 0.247 mmol/L, respectively, all of which were statistically significant (*p* < 0.05). See Table 3 for details.

#### 3.6.3 Nutritional intervention

Among nutritional intervention indicators, compared to patients who did not receive any intervention, those who received enteral nutrition, parenteral nutrition, and combined nutrition had reductions in HDL-C levels by 0.050 mmol/L, 0.290 mmol/L, and 0.215 mmol/L, respectively. Adjustments for confounding factors did not alter these results, and the differences among the indicators were not statistically significant (all *P*s > 0.05).

## 4 Discussion

Low HDL-C levels are an important risk factor for cardiovascular and cerebrovascular diseases, and the prevalence remains high among Chinese residents. This study comprehensively investigated the HDL-C levels of newly hospitalized adult patients in Jiangsu Province. The results indicate that the prevalence of low HDL-C levels in newly hospitalized patients in Jiangsu Province is as high as 30.7%, urgently requiring attention.

This study indicated that men were more likely than women to have low HDL-C levels, which is in line with research conducted in China by Pan et al. (15) but differs from some international studies. For example, Mohamud et al.’s study in Malaysia showed a lower prevalence in men compared to women (32.6% vs. 48.1%) (16). A similar trend was observed in an Iranian study (29.4% vs. 54.7%). Nevertheless, the average HDL-C level in men (1.083 mmol/L) remained lower than in women (1.175 mmol/L) (17). The differences in research results at home and abroad may be attributed to the varying definitions of low HDL-C criteria between domestic and international studies. Many foreign studies adopt the National Cholesterol Education Program Adult Treatment Panel III criteria (18) and the International Diabetes Federation (19) standards (men <1.0 mmol/L, women <1.3 mmol/L), whereas domestic studies apply a uniform threshold of <1.0 mmol/L regardless of sex. The stricter criterion for women in domestic studies might lead to an underestimation of the prevalence of low HDL-C in women, while the higher diagnostic threshold (1.3 mmol/L) in foreign studies results in a relatively higher prevalence in women. This is an important reason for the inconsistency in findings across studies.

Our study shows that the prevalence of low HDL-C followed an “N” shaped distribution with age, increasing, then decreasing, and finally increasing again. The age group of 90–99 years had the highest risk, suggesting that low HDL-C might be an age-related degenerative condition. This finding contrasts with some studies. For instance, the study by Latifi et al. (17), which involved 2,505 adults over 20 in Ahvaz, Iran, found no correlation between the prevalence of low HDL-C levels and aging. In contrast, Erem et al. (20) reported that in Trabzon, Turkey, the prevalence first increased and then decreased with age, a difference possibly due to the lack of detailed age groupings above 70 and insufficient samples for those over 80 in those studies. Further research is needed to explore these discrepancies.

Hospitals commonly use the NRS2002, developed by Kondrup et al., as a nutritional risk screening tool. This tool considers changes in food intake and disease severity (21). Its identification of “nutritional risk” is closely linked to clinical outcomes, making it the preferred screening tool in many guidelines (22). Our study demonstrated that patients with nutritional risks exhibited a higher propensity for reduced HDL-C levels than those without such risks. This association may be attributable to the heightened inflammatory state frequently observed in individuals with nutritional deficiencies. Our research confirmed the association of severe infections, fever, high CRP, and hypoalbuminemia with lower HDL-C levels. Malnutrition or nutritional risk states can also lead to lipid metabolism abnormalities (23), affecting HDL-C production and metabolism. Thus, patients with nutritional risks have a higher risk of low HDL-C, consistent with our findings.

An additional nutritional assessment is required for individuals with a screening total score of 3 or above, as screening attempts to determine risk while evaluation elucidates nutritional status (24). This study demonstrates that individuals with constipation exhibit an elevated risk of low HDL-C compared to those without constipation. Poor diets that include high-fat, low-fiber meals, problems with nutrient absorption, and an imbalance in the gut microbiota may all be associated with this risk. For constipated people to improve their nutrition and cholesterol levels, comprehensive therapies involving food, lifestyle modifications, and medication are required. Low HDL-C is less likely to occur in patients with esophageal stricture, according to the study. This conclusion might be explained by the fact that individuals with esophageal stricture typically choose high-nutrient, high-energy liquid meals because they have trouble eating. HDL-C levels are raised by these diets, which are high in vitamins, minerals, and vital fatty acids. Essential fatty acids, particularly *ω*-3 fatty acids, have been demonstrated to increase HDL-C levels (6). These patients are periodically given dietary recommendations or nutritional interventions, which help to detect and promptly treat any potential nutritional deficiencies.

According to this study, individuals with inflammation or disease burden had fever, high CRP, hypoalbuminemia, and severe infections; these symptoms are linked to reduced HDL-C values. This finding aligns with both domestic and international research. In infectious diseases, in addition to inflammatory factors, lipid metabolism, particularly HDL-C and LDL-C, as well as protein metabolism, also contribute to disease progression. HDL-C has anti-inflammatory and antioxidant properties (25) and may be substantially depleted or inhibited during infection. HDL-C significantly enhances the host’s resistance to bacterial, viral, and parasitic infections, suggesting its active role in innate immune responses (26). Observational studies show that HDL-C is linked to a lower risk of future infections. HDL contains proteins that help activate the complement system and control inflammation (27). Further studies show that low HDL-C levels at admission are linked to a higher risk of infections during hospital stays (28, 29). These findings support our conclusion that patients with severe infections have a higher risk of low HDL-C levels than those without infections. Elevated CRP levels, an inflammation marker, usually indicate acute or chronic inflammatory states. Fever represents a physiological stress response to infection or inflammation, often associated with elevated CRP levels and a concomitant reduction in HDL-C levels. Rashidi et al. (6) found a strong link between serum albumin and HDL-C levels, suggesting that low albumin increases the risk of low HDL-C. This relationship may be attributed to the multifaceted roles of albumin. This most abundant plasma protein maintains colloidal osmotic pressure, facilitating molecular transport, providing antioxidative and anti-inflammatory effects, preventing thrombosis, and regulating capillary permeability. Hypoalbuminemia can change plasma properties, affecting HDL structure and function (30). Apolipoprotein A1 (ApoA1) is the main protein in HDL and is important for cholesterol transport and lipid metabolism. Low protein levels, or hypoproteinemia, can increase protein breakdown, such as via the ubiquitin-proteasome system, quickly degrading proteins like ApoA1, which disrupts lipid metabolism and lowers HDL-C levels (31). The liver creates and breaks down lipoproteins. Less albumin from the liver can indicate liver issues or metabolism changes, affecting lipoprotein metabolism and reducing HDL-C levels (32).

Our study results show that CKD patients have a higher risk of low HDL-C. Changes in serum lipid profiles, redox status, and inflammatory markers are intimately linked to CKD, which may explain these phenomena (33). Due to several factors, HDL’s protective role is diminished in CKD patients, and HDL-related enzyme activity, such as paraoxonase, is decreased (34). As a result, the antioxidant protective properties of HDL isolated from CKD patients are significantly diminished, leaving them more vulnerable to infections and inflammation. HDL from kidney disease patients offers less protection against oxidative stress, increasing their risk of inflammation and infection. A study found a U-shaped relationship between HDL-C levels and the risk of kidney disease in 1,943,682 men in the U. S. (35). Veterans. Conversely, Caroline et al. (36) found that higher HDL-C levels were linked to less kidney disease progression in 2,585 U. S. adults. The difference from our study may be because we did not have enough new patients with high HDL-C levels to see the relationship. This study indicates that patients with a history of tumors have a lower risk of low HDL-C levels. This conclusion is not entirely consistent with existing studies, both domestically and internationally. Many studies currently evaluate the link between HDL-C levels and the risk of different cancers. For instance, Alicia et al. (37) conducted a Mendelian randomization analysis involving 181,677 European women and found a positive correlation between HDL-C levels and breast cancer risk. In contrast, a large prospective cohort study from Japan (38) reported opposing findings.

Additionally, Jennifer et al. (39) observed no association between HDL-C levels and breast cancer risk in a prospective study of Swedish women aged 25 and above. The discrepancy in conclusions may be attributed to the prospective nature of these studies. Our analysis shows that tumor patients have relatively higher HDL-C levels, which might be related to the sampling healthcare institutions—patients at these institutions are often not in the initial diagnosis stage and have received relevant interventions and treatments. Many cancer patients adjust their lifestyle post-diagnosis, such as quitting smoking, losing weight, improving diet, and increasing physical activity, all of which can elevate HDL-C levels. Furthermore, the tumor and its treatment can activate or inhibit different inflammatory and immune responses, indirectly affecting lipid metabolism. Future research should check if new cancer patients also have low HDL-C levels like we found.

For patients diagnosed with malnutrition after screening and assessment, nutritional interventions are typically implemented. These interventions mainly include enteral nutrition support, parenteral nutrition support, or a combined nutrition support regimen of both. Our study shows that patients on parenteral nutrition often have lower HDL-C levels and a higher risk of low HDL-C. This observation might be explained by the fact that patients requiring parenteral nutrition typically present with more severe clinical conditions or critical illnesses. These states are strongly associated with heightened systemic inflammation and oxidative stress, disrupting normal lipoprotein metabolism. High inflammation markers like CRP can reduce HDL-C production and its function, raising the risk of low HDL-C levels. Parenteral nutrition frequently contains high-fat emulsions, particularly those made from soybean oil, which are high in *ω*-6 polyunsaturated fatty acids and long-chain triglycerides, which are known to have pro-inflammatory effects (40, 41). In addition, several studies have indicated that parenteral nutrition therapy may be associated with excessive increases in glucose and lipid loads. These overload conditions may trigger hyperglycemia, pro-inflammatory responses, abnormal white blood cell function, endothelial dysfunction, and exacerbated oxidative stress in some patients (42, 43). These factors may indirectly contribute to the formation of low HDL-C levels by affecting the physiological pathways of lipoprotein metabolism.

In light of our findings, hospitals could consider incorporating HDL-C levels as a supplemental indicator in nutritional assessments and intervention prioritization. Given the strong association between nutritional risk and reduced HDL-C levels, routine screening for HDL-C could help identify patients at higher risk of malnutrition or adverse clinical outcomes. This approach might enhance the precision of nutritional risk profiling, allowing for more targeted interventions. For example, patients with low HDL-C levels could be prioritized for comprehensive nutritional evaluations and personalized interventions aimed at improving dietary quality, promoting physical activity, and addressing underlying inflammatory conditions.

The advantages of our study are as follows. First, to our knowledge, there has been limited research on the changes in HDL-C levels and their clinical significance in hospitalized patients with nutritional risk who have undergone nutritional assessment and intervention. Our study is one of the first to demonstrate an association between integrated nutritional risk screening and nutritional interventions with changes in HDL-C levels among newly admitted patients, highlighting their clinical significance. Second, our research is a cross-sectional multicenter study with a large sample size, providing higher representativeness and generalizability. Finally, our study accounted for potential confounding variables, including demographic characteristics and comorbidities.

However, this study has limitations. Because of the cross-sectional nature of this study, we were unable to infer causal relationships between nutritional risk, interventions, and HDL-C levels. While associations were observed, these relationships may be bidirectional or confounded by factors such as disease severity, inflammation, and comorbidities. Further longitudinal or interventional studies are warranted to establish causal pathways. Additionally, due to the exclusion of hospitalized patients with mental disorders, memory impairments, critical illnesses, and those lacking behavioral capabilities, the sample may have selection bias.

## 5 Conclusion

In summary, the prevalence of low HDL-C in newly admitted patients in Jiangsu Province is high, exhibiting an “N-shaped” distribution with age. Factors associated with lower HDL-C levels include nutritional risk, nausea, vomiting, constipation, pancreatic insufficiency, severe infections, chronic kidney disease, fever, high CRP, hypoalbuminemia, and parenteral nutrition. Conversely, having esophageal stricture, cancer, or head injury is linked to higher HDL-C levels. This finding helps guide nutritional treatments and manage chronic diseases.

## Data Availability

The raw data supporting the conclusions of this article will be made available by the authors, without undue reservation.
