# Supplementary material for: Relationship between high-density lipoprotein cholesterol levels and nutritional risk screening-assessment-intervention: a multicenter cross-sectional study
Source: Front Nutr. 2025 Jun 18;12:1528068. doi: 10.3389/fnut.2025.1528068 (PMC12213356; doi:10.3389/fnut.2025.1528068)
Supplement: Supplementary file 1 [file Table_1.pdf]

Supplementary materials

Table 1 The levels of HDL-C (mmol/L) in newly hospitalized patients and the nutritional risk characteristics of the general population in 23 hospitals across 12 cities in Jiangsu Province.

Characteristics	N	HDL-C < 1 N(%)	HDL-C ≥1 N(%)	$\chi 2$	P	HDL concentration P_{50} ($P_{25\sim}$ P_{75})	P	N	No nutritional risk N(%)	nutritional risk N(%)	$\chi 2$	P
Diagnosis of Eye, Ear, Nose, and Throat Diseases												
No	4151	1274(30.7%)	2877(69.3%)	0.127	0.722	1.120(0.870-1.440)	0.719	4546	3908(86.0%)	638(14.0%)	0.885	0.347
Yes	39	13(33.3%)	26(66.7%)			1.110(0.843-1.320)		44	40(90.9%)	4(9.1%)		
Diagnosis of Skin and Subcutaneous Tissue Diseases												
No	4171	1281(30.7%)	2890(69.3%)	0.007	0.935	1.120(0.870-1.440)	0.620	4569	3930(86.0%)	639(14.0%)	0.002	0.968
Yes	19	6(31.6%)	13(68.4%)			1.108(0.830-1.743)		21	18(85.7%)	3(14.3%)		
Diagnosis of Musculoskeletal System Diseases												
No	4142	1275(30.8%)	2867(69.2%)	0.745	0.388	1.120(0.870-1.440)	0.115	4536	3901(86.0%)	635(14.0%)	0.048	0.827
Yes	48	12(25.0%)	36(75.0%)			1.285(0.943-1.621)		54	47(87.0%)	7(13.0%)		
Diagnosis of Immune System Diseases												
No	4167	1278(30.7%)	2889(69.3%)	0.769	0.380	1.120(0.870-1.440)	0.295	4566	3928(86.0%)	638(14.0%)	0.144	0.704
Yes	23	9(39.1%)	14(60.9%)			1.125(0.615-1.310)		24	20(83.3%)	4(16.7%)		
Diagnosis of complications during pregnancy, childbirth, and the postpartum period												
No	4186	1286(30.7%)	2900(69.3%)	0.061	0.804	1.120(0.870-1.440)	0.912	4586	3944(86.0%)	642(14.0%)	0.651	0.420
Yes	4	1(25.0%)	3(75.0%)			1.110(-1.705-2.699)		4	4(100%)	0		
Diagnosis of Malnutrition-Related Diseases												
No	4172	1278(30.6%)	2894(69.4%)	3.159	0.076	1.120(0.870-1.440)	0.336	4572	3934(86.0%)	638(14.0%)	1.019	0.313
Yes	18	9(50.0%)	9(50.0%)			1.030(0.623-1.423)		18	14(77.8%)	4(22.2%)		

The bold values are statistically significant, with *p*-values all less than 0.05. In addition, the numbers in bold use the same statistical method. Mann Whitney U Nonparametric Test or K Independent Samples Median Nonparametric Test was used to describe median differences by continuous variables and the chi-square test was used to examine differences in categorical variables.

Table 2 The relationship between HDL-C levels (mmol/L) and nutritional risk screening, assessment, and intervention in newly hospitalized patients in 23 hospitals across 12 cities in Jiangsu Province.

Characteristics	N	HDL-C < 1 N(%)	HDL-C ≥1 N(%)	χ2	P	HDL concentration P50 (P25~ P 75)	P	N	No nutritional risk N(%)	nutritional risk N(%)	χ2	P
Gastroparesis												
Yes	4	1(25.0%)	3(75.0%)	0.062	0.804	1.575(0.990-3.478)	0.173	4	3(75.0%)	1(25.0%)	0.403	0.525
No	4185	1286(30.7%)	2899(69.3%)			1.120(0.870-1.440)		4585	3944(86.0%)	641(14.0%)		
Steatorrhea												
Yes	8	4(50.0%)	4(50.0%)	1.399	0.237	0.965(0.288-1.330)	0.383	8	7(87.5%)	1(12.5%)	0.015	0.903
No	4181	1283(30.7%)	2898(69.3%)			1.120(0.870-1.440)		4581	3940(86.0%)	641(14.0%)		
Chronic Liver Disease												
Yes	107	41(38.3%)	66(61.7%)	2.975	0.085	1.110(0.780-1.350)	0.224	115	95(82.6%)	20(17.4%)	1.134	0.287
No	4082	1246(30.5%)	2836(69.5%)			1.120(0.880-1.440)		4474	3852(86.1%)	622(13.9%)		

The bold values are statistically significant, with p-values all less than 0.05. In addition, the numbers in bold use the same statistical method. Mann Whitney U Nonparametric Test or K Independent Samples Median Nonparametric Test was used to describe median differences by continuous variables and the chi-square test was used to examine differences in categorical variables.

Table 3 The unadjusted and adjusted odds ratios (95% CIs) and regression coefficients (95% CIs) for HDL-C levels and nutritional risk screening, assessment, and intervention in newly hospitalized patients.

Characteristics	N	OR (95%CI)	*P*	Adjusted OR (95%CI)	*P*	β (95%CI)	*P*	Adjusted β (95%CI)	*P*
Gastroparesis									
Yes	4	0.751(0.078,7.231)	0.805	0.560（0.058,5.441）	0.617	0.817(-0.445,2.080)	0.204	0.819（-0.377,2.014）	0.179
No	4185	1(ref)							
Steatorrhea									
Yes	8	2.259(0.564,9.046)	0.250	2.398（0.584,9.849）	0.225	-0.437(-1.330,0.456)	0.338	-0.465（-1.311,0.382）	0.282
No	4181	1(ref)							

Chronic Liver Disease									
Yes	107	1.414(0.952,2.100)	0.086	1.205(0.772, 1.881)	0.412	-0.084(-0.322,0.155)	0.491	0.008（-0.239,0.255）	0.949
No	4082	1(ref)							

The bold values are statistically significant, with *p*-values all less than 0.05. In addition, the bold values use the same statistical method. Linear regression was used to assess the linear relationship between HDL-C levels and nutritional risk screening, evaluation, and intervention. Binary logistic regression was used to evaluate the association of these factors with dichotomous outcomes related to HDL-C levels.The adjusted indicators include age, sex, patient education level, regional distribution, diagnosis of infectious and parasitic diseases, diagnosis of tumors, diagnosis of diseases of the blood and blood-forming organs, diagnosis of endocrine, nutritional, and metabolic diseases, diagnosis of circulatory system diseases, diagnosis of genitourinary system diseases, and the number of diagnosed diseases, in addition to the unadjusted basis.
